# Metagenomic next-generation sequencing for the diagnosis of pulmonary aspergillosis in non-neutropenic patients: a retrospective study

**DOI:** 10.3389/fcimb.2022.925982

**Published:** 2022-08-01

**Authors:** Shujun Bao, Huihui Song, Yang Chen, Caiming Zhong, Hao Tang

**Affiliations:** Department of Respiratory and Critical Care Medicine, Second Affiliated Hospital of Naval Medical University, Shanghai, China

**Keywords:** metagenomic next-generation sequencing, aspergillus, pulmonary aspergillosis, non-neutropenic patients, diagnosis

## Abstract

This study aimed to obtain further in-depth information on the value of metagenomic next-generation sequencing (mNGS) for diagnosing pulmonary aspergillosis in non-neutropenic patients. We did a retrospective study, in which 33 non-neutropenic patients were included, of which 12 were patients with pulmonary aspergillosis and 21 were diagnosed with non-pulmonary aspergillosis. Fungi and all other co-pathogens in bronchoalveolar lavage fluid (BALF) (27 cases), blood (6 cases), and/or pleural fluid (1 case) samples were analyzed using mNGS. One of the patients submitted both BALF and blood samples. We analyzed the clinical characteristics, laboratory tests, and radiologic features of pulmonary aspergillosis patients and compared the diagnostic accuracy, including sensitivity, specificity, positive predictive value, and negative predictive value of mNGS with conventional etiological methods and serum (1,3)-β-D-glucan. We also explored the efficacy of mNGS in detecting mixed infections and co-pathogens. We further reviewed modifications of antimicrobial therapy for patients with pulmonary aspergillosis according to the mNGS results. Finally, we compared the detection of *Aspergillus* in BALF and blood samples from three patients using mNGS. In non-neutropenic patients, immunocompromised conditions of non-pulmonary aspergillosis were far less prevalent than in patients with pulmonary aspergillosis. More patients with pulmonary aspergillosis received long-term systemic corticosteroids (50% vs. 14.3%, p < 0.05). Additionally, mNGS managed to reach a sensitivity of 91.7% for diagnosing pulmonary aspergillosis, which was significantly higher than that of conventional etiological methods (33.3%) and serum (1,3)-β-D-glucan (33.3%). In addition, mNGS showed superior performance in discovering co-pathogens (84.6%) of pulmonary aspergillosis; bacteria, bacteria-fungi, and bacteria-PJP-virus were most commonly observed in non-neutropenic patients. Moreover, mNGS results can help guide effective treatments. According to the mNGS results, antimicrobial therapy was altered in 91.7% of patients with pulmonary aspergillosis. The diagnosis of *Aspergillus* detected in blood samples, which can be used as a supplement to BALF samples, seemed to show a higher specificity than that in BALF samples. mNGS is a useful and effective method for the diagnosis of pulmonary aspergillosis in non-neutropenic patients, detection of co-pathogens, and adjustment of antimicrobial treatment.

## Introduction

*Aspergillus* is one of the most ubiquitous and important pathogens in the environment, including *Aspergillus fumigatus*, *A. flavus*, *A. niger*, *A. terreus*, *A. nidulans*, and others (Sugui et al., 2014). *Aspergillus*-related lung diseases are traditionally classified into four types: pulmonary aspergilloma, allergic broncho-pulmonary aspergillosis (ABPA), chronic pulmonary aspergillosis (CPA), and invasive aspergillosis (IPA) (Chabi et al., 2015). Aspergillus occurrence and clinical manifestations depend on the host’s immunological status and the existence of underlying lung disease. Recently, researchers have discovered that aspergillosis can arise in other diseases, such as long-term corticosteroid use in chronic obstructive pulmonary disease (COPD) (Patterson and Strek, 2014), diabetes mellitus, pulmonary tuberculosis, and bronchiectasis, rather than in immunocompromised hosts. Even without disease, potential hosts can be attacked when they are in contact with a large mass of *Aspergillus*.

The clinical manifestations of pulmonary aspergillosis in non-neutropenic patients are non-specific, even similar to tuberculosis (TB), which makes diagnosis difficult. Non-neutropenic patients who are immunocompromised owing to other factors have a more progressive course and a worse prognosis than the neutropenic patients. Thus, recognizing the diversity and subtle representation of the disease, assessing vulnerable groups, and using effective therapy sooner in the disease stage are very valuable.

To date, the definitive diagnostic method of pulmonary aspergillosis is a pathological investigation that takes too much time (Li, 2022). Conventional etiological methods, namely fungal smear and culture, are time-consuming and show a low detection rate. Recently, non-invasive biomarkers have made it easier to suspect and diagnose pulmonary aspergillosis (El-Baba et al., 2020). However, serum (1,3)-β-D-glucan (G) or galactomannan (GM) test results should be considered in combination with clinical manifestations in pediatric pulmonary aspergillosis cases, as the tests are not sufficiently sensitive (Tong et al., 2018). The introduction of several high-performing diagnostic tests helps redefine patient management. Researchers have suggested that *Aspergillus* antigen and *Aspergillus* immunoglobulin G (precipitins) are promising markers for the diagnosis of *Aspergillus* (Denning, 2021). However, there is an urgent need to explore new and efficient (quicker and more accurate) diagnostic tools for pulmonary aspergillosis to acquire early identification, improve patient outcomes, and reduce mortality.

Based on high-throughput sequencing, metagenomic next-generation sequencing (mNGS) is thought to be a promising microbial identification technology because of its rapid cycles and high sensitivity. mNGS identifies and classifies a wide range of pathogens (including respiratory tract (Xie et al., 2021), blood stream (Jing et al., 2021), central nervous system (Wilson et al., 2019), and prosthetic joints pathogens (Thoendel et al., 2018)), and is a widely used microbial test for infectious diseases, particularly for special and rare pathogens. It can also be used to analyze drug resistance genes and virulence factors in pathogens.

Many previous studies on the detection of pulmonary infections using mNGS have focused on pathogens. Yang et al. (Yang et al., 2021) observed that mNGS (lung biopsy, bronchoalveolar lavage fluid [BALF]) had a significantly higher sensitivity than conventional tests for diagnosing pulmonary fungal infections. Liu et al. (Liu et al., 2021) evaluated the performance of BALF-mNGS in differentiating colonization and infection with *Pneumocystis jirovecii* and suggested that the fungal load differed significantly between the two groups (*P. jirovecii* pneumonia and *P. jirovecii* colonization). However, there is still a scarcity of clinical experience in the diagnosis of fungal infections, particularly aspergillosis.

Therefore, our retrospective study aimed to contribute to this growing area of research by exploring the diagnostic performance of mNGS in non-neutropenic patients with pulmonary aspergillosis.

## Materials and methods

### Study design and subjects

In this retrospective study, we successively enrolled 33 non-neutropenic patients whose mNGS results (BALF and/or blood and/or pleural fluid samples) identified fungi (e.g., *Aspergillus*, *P. jirovecii*, *Candida*, and *Cryptococcus*) and were admitted to the Department of Respiratory and Critical Care Medicine of Second Affiliated Hospital, Naval Medical University (Shanghai, China), from June 1, 2018, to January 31, 2022. The combined clinical diagnosis of pulmonary aspergillosis or non-pulmonary aspergillosis was made by two medical doctors based on host risk factors, clinical symptoms, chest computed tomography images, laboratory findings, and response to treatment. Finally, 12 patients were diagnosed with pulmonary aspergillosis, and 21 were diagnosed with non-pulmonary aspergillosis. Patients were excluded from the study based on the following criteria: (1) age <18 years; (2) neutropenia (absolute neutrophil count <1.5×10^9^/L); (3) mNGS not performed; and (4) incomplete medical records.

### The collection of biological samples

Collection of BALF was performed by experienced bronchoscopists at Naval Medical University’s Second Affiliated Hospital after treatment with atropine for spasmolysis, diazepam for sedation, and lidocaine for local anesthesia. The sampling site was chosen based on chest computed tomography (CT) images. Three 20 ml sterile saline fractions were instilled into the target subsegmental bronchi. BALF was extracted using gentle syringe suction and was placed in sterile containers. To avoid contamination, the first 20 ml of each sample was discharged, while the rest samples were kept for analysis. Furthermore, 3–5 ml of blood samples and at least 10 ml pleural fluid samples were collected.

### Testing process of mNGS

DNA extraction and sequencing were conducted by the Beijing Genomics Institute (BGI). First, BALF was mixed with lysozyme and glass beads to reduce viscosity. Blood was centrifuged to separate the plasma, and pleural fluid was centrifuged to collect the sediment. The inactivated specimens were further subjected to wall breaking. Following this, DNA was extracted. The concentration and volume of the extracted samples were measured to determine the quality control results of the nucleic acids. Construction of DNA library and sequencing the DNA library was done by DNA fragmentation, end-repair, and polymerase chain reaction (PCR) amplification. This was then used for library concentration detection and quality control. Qualified DNA nanoballs were loaded onto the chip, and single-end sequencing was performed on the MGISEQ-2000 sequencing platform. Bioinformatic analysis was conducted on samples with raw data >20 million and read length >50bp (Long et al., 2016). Low-quality human host sequences were removed (Marotz et al., 2018) to improve detection sensitivity (Hasan et al., 2016). The high-quality data were classified by simultaneously aligning to microbial genome databases (Salter et al., 2014), including viruses, bacteria, fungi, and parasites. The database reference sequences were used to calculate the microbial parameters *via* multiple comparisons with de-exclusion. Eventually, clinically significant microbes were categorized as putative co-pathogens if the patients had suggestive symptoms, laboratory findings, and radiologic abnormalities.

### Statistical analysis

Statistical analysis was performed using the SPSS software (version 26, IBM Corp, Armonk, NY, USA). Continuous variables were presented as mean ± standard deviation, whereas categorical variables were presented as counts and percentages. The t-test for two independent samples was used to compare the normal distribution of continuous variables between pulmonary aspergillosis and non-pulmonary aspergillosis patients, the Mann–Whitney U test was used to compare the abnormal distribution of continuous variables, and the chi-square test was used for categorical variables. The sensitivity, specificity, positive predictive value (PPV), and negative predictive value (NPV) of mNGS, conventional etiological methods, and serum (1,3)-β-D-glucan in the diagnosis of pulmonary aspergillosis were calculated using the combined clinical diagnosis as the reference standard; Wilson’s method was used to calculate 95% confidence intervals for these proportions. Statistical significance was set at P < 0.05.

### Ethics statement

Ethical approval was obtained from the Ethics Committee of Shanghai Changzheng Hospital. The patients provided their written informed consent to participate in this study.

## Results

### Clinical characteristics, laboratory tests, and radiologic features of pulmonary aspergillosis in non-neutropenic patients

**Table 1** summarizes the patient characteristics on admission. The cohort was divided into two groups according to clinical composite diagnosis. A total of 12 pulmonary aspergillosis patients and 21 non-pulmonary aspergillosis patients were included in this study. The median age (60 vs. 68 years) and sex composition of these two groups were similar. The most prevalent symptoms of pulmonary aspergillosis in non-neutropenic patients were cough (91.7%), expectoration (75.0%), fever (75.0%), and abnormal breath sounds (90.0%). Comparing the two results, chest tightness and dyspnea were less frequently observed in patients with pulmonary aspergillosis. Among the underlying diseases of pulmonary aspergillosis, diabetes (50.0%) ranked first, followed by malnutrition (41.7%), COPD (25.0%), and solid tumors (25.0%). In addition, the data showed that patients with pulmonary aspergillosis had various immunocompromised conditions: 50.0% received long-term systemic corticosteroids (used for at least 3 weeks with a daily dose of ≥0.3mg/kg/day of prednisone or the equivalent dose of prednisone) (p = 0.044), 41.7% received cytotoxic drugs (within 90 days), 33.3% received prior broad-spectrum antibiotics, and 25.0% received immunosuppressive agents (within 90 days). In contrast, immunocompromised conditions were far less prevalent in the non-pulmonary aspergillosis patients.

**Table 1:** Clinical characteristics, laboratory tests, and radiologic features of pulmonary aspergillosis and non-pulmonary aspergillosis in non-neutropenic patients on admission.

Characteristic (mean± [standard deviation] or count [percentage])	Pulmonary aspergillosis patients(n = 12)	Non-pulmonary aspergillosis patients(n = 21)	P-value
Male	7 (58.3%)	17 (81%)	0.230
Age (years)	60.3 ± 14.9	67.8 ± 17.4	0.399
Smoking history	4 (33.3%)	9 (42.9%)	0.719
At least two hospitalization times per year	6 (50.0%)	7 (33.3%)	0.465
Abnormal environmental exposure history	0 (0%)	1 (4.8%)	1.000
Less than 2 weeks after onset	7 (58.3%)	15 (71.4%)	0.471
Mechanical ventilation	3 (25%)	5 (23.8%)	1.000
Hemodynamic instability	0 (0%)	4 (19%)	0.271
**Clinical symptoms**			
Fever	9 (75%)	14 (66.7%)	0.710
Cough	11 (91.7%)	17 (81%)	0.630
Expectoration	9 (75%)	14 (66.7%)	0.710
Hemoptysis	3 (25%)	3 (14.3%)	0.643
Chest tightness/dyspnea	7 (58.3%)	13 (61.9%)	1.000
Abnormal breath sound	9 (90% [n=10])	18 (90% [n=20])	1.000
**Underlying diseases**			
Bronchiectasis	1 (8.3%)	6 (28.6%)	0.223
Interstitial lung disease	2 (16.7%)	2 (9.5%)	0.610
Emphysema/COPD	3 (25%)	2 (9.5%)	0.328
Tuberculosis	0 (0%)	3 (14.3%)	0.284
Malnutrition	5 (41.7%)	6 (28.6%)	0.471
Diabetes	6 (50%)	4 (19%)	0.114
Solid tumors	3 (25%)	6 (28.6%)	1.000
**Immunocompromised conditions**			
With HIV-infected	0 (0%)	2 (9.5%)	0.523
Systemic use of corticosteroids	6 (50%)	3 (14.3%)	0.044^***^
Use of immunosuppressive agents	3 (25%)	2 (9.5%)	0.328
Use of cytotoxic drugs	5 (41.7%)	2 (9.5%)	0.071
Use of prior broad-spectrum antibiotics	4 (33.3%)	3 (14.3%)	0.377
**Chest computed tomography images**			
Double lung infiltration	11 (91.7%)	17 (81%)	0.630
Multiple lesions	11 (91.7%)	18 (85.7%)	1.000
Aspergilloma	1 (8.3%)	0 (0%)	0.364
Nodular shadowing	4 (33.3%)	6 (28.6%)	1.000
Wedge or patchy shadowing	8 (66.7%)	19 (90.5%)	0.159
Cavitation sign	2 (16.7%)	0 (0%)	0.125
Crescent sign	0 (0%)	0 (0%)	NaN
Halo sign	0 (0%)	0 (0%)	NaN
Pleural effusion	7 (58.3%)	14 (58.3%)	0.716
Mediastinal lymphadenopathy	3 (25%)	6 (28.6%)	1.000
Thickening of the bronchial lumen	1 (8.3%)	6 (28.6%)	0.223
Abnormal changes under endoscopy	5 (71.4% [n=7])	11 (73.3%[n=15])	1.000
**Laboratory findings**			
WBC (×10^9/L)	13.2 ± 11.3	9.7 ± 3.1	0.837
Neu (%)	82.1 ± 10.6	81.3 ± 9.2	0.837
CRP (mg/L)	62.6 ± 53.5	70.5 ± 67.6	0.940
PCT (>0.5 ng/ml)	4 (36.4% [n=11])	5 (23.8% [n=21])	0.681
ESR (>15 mm/h)	8 (80% [n=10])	13 (92.9% [n=14])	0.550
ALB (g/L)	29.4 ± 4.9	33.3 ± 4.8	0.447
Glucose control (HbA1c>6.5%)	4 (57.1% [n=7])	3 (50% [n=6])	1.000
D-dimers (>0.55 ug/ml)	11 (100% [n=11])	13 (72.2% [n=18])	0.126
Type I respiratory failure (<60 mmHg)	3 (42.9% [n=7])	5 (45.5% [n=11])	1.000
Immunosuppression (CD4/CD8 ratio<1.4)	5 (62.5% [n=8])	7 (50% [n=14])	0.675
Serum (1,3)-β-D-Glucan (>100 pg/ml)	4 (36.4% [n=11])	3 (15.8% [n=19])	0.372

HIV, human immunodeficiency virus; WBC, white blood cell; Neu, neutrophils; CRP, C-reactive protein; PCT, procalcitonin; ESR, erythrocyte sedimentation rate; ALB, albumin.

^***^P<0.05.

**Figure 1:**
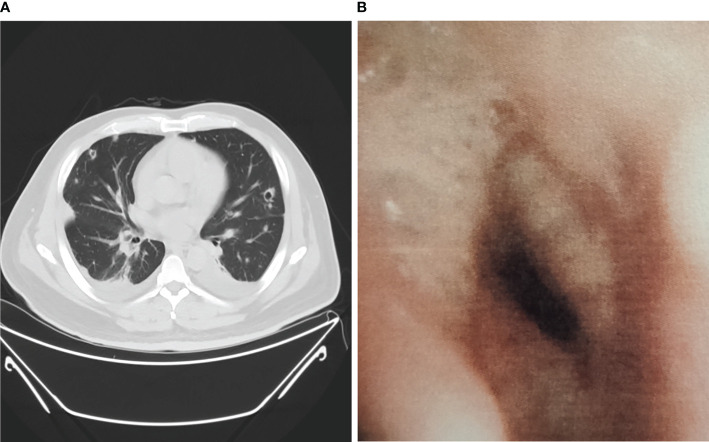
Chest CT scan and bronchoscopy of pulmonary aspergillosis in non-neutropenic patient. **(A)** Chest CT showed cavitation signs; **(B)** Bronchoscopy showed purulent secretions, mucosal hyperemia, and edema.

Among non-neutropenic patients with pulmonary aspergillosis, the most common radiologic features on chest CT were double lung infiltration (91.7%), multiple lesions (91.7%), wedge or patchy shadowing (66.7%), and nodular shadowing (33.3%). Some showed typical features such as aspergilloma (8.3%) and cavitation signs (16.7%) (**Figure 1A**), which may be accompanied by pleural effusion, mediastinal lymphadenopathy, and abnormal changes during endoscopy (**Figure 1B**). The next section of the study was concerned with the laboratory findings. Serum (1,3)-β-D-glucan levels, d-dimer levels, immunosuppression, and poor glucose control in pulmonary aspergillosis patients appeared to be higher than those in non-pulmonary aspergillosis patients, but there were no significant differences.

### Comparison of diagnostic performance among mNGS, conventional etiological methods, and serum (1,3)-β-D-glucan in non-neutropenic pulmonary aspergillosis patients

One of the most well-known tools for assessing *Aspergillus* infection is the conventional etiological method; serum (1,3)-β-D-glucan and galactomannan are widely used serologic biomarkers of pulmonary aspergillosis. Recently, molecular biological techniques such as mNGS have been widely applied in clinical settings. Therefore, we compared the diagnostic accuracy of mNGS with that of conventional etiological methods and serum (1,3)-β-D-glucan. **Table 2** shows it that mNGS of BALF, blood, and/or pleural fluid samples was performed in all 34 cases, conventional etiological methods in 31 cases (12 with pulmonary aspergillosis and 19 with non-pulmonary aspergillosis) and serum (1,3)-β-D-glucan in 31 cases (12 with pulmonary aspergillosis and 19 with non-pulmonary aspergillosis). The combined clinical diagnosis was used as reference to calculate the diagnostic sensitivity and specificity of mNGS. The sensitivities of mNGS, conventional etiological methods, and serum (1,3)-β-D-glucan were 91.7% (95% CI, 59.8–99.6%), 33.3% (95% CI, 11.3–64.6%), and 33.3% (95% CI, 11.3–64.6%), respectively. However, mNGS showed a low specificity of 71.4% (95% CI, 47.7–87.8%), compared with conventional etiological methods (100% [95% CI, 79.1–100%]) and serum (1,3)-β-D-glucan (84.2% [95% CI, 59.5–95.8%]). With the highest Youden index of 0.63, mNGS appears to have the most effective screening ability and the best authenticity.

**Table 2:** Diagnostic performance of mNGS, conventional etiological methods, and serum (1,3)-β-D-glucan in non-neutropenic pulmonary aspergillosis patients.

**Number of cases**	**Methods**	**Pulmonary aspergillosis cohort**	**Non-pulmonary aspergillosis cohort**	**Sensitivity (95% CI)**	**Specificity (95% CI)**	**PPV (95% CI)**	**NPV (95% CI)**	**Youden index**
n = 34	mNGS+	11	6	91.7% (0.598–0.996)	71.4% (0.477–0.878)	64.7% (0.386–0.847)	93.8% (0.677–0.997)	0.63
	–	1	15					
n = 31	Conventional etiological methods+	4	0	33.3% (0.113–0.646)	100% (0.791–1)	100% (0.396–1)	70.4% (0.497–0.855)	0.33
	–	8	19					
n = 31	G test+	4	3	33.3% (0.113–0.646)	84.2% (0.595–0.958)	57.1% (0.202–0.882)	66.7% (0.447–0.836)	0.18
	–	8	16					

Aspergillus detected by mNGS, Aspergillus detected by conventional etiological methods, and serum (1,3)-β-D-glucan level > 100 pg/ml were defined as positive.

PPV, positive predictive value; NPV, negative predictive value; CI, confidence interval; G, serum (1,3)-β- D-glucan.

### Mixed infections and co-pathogens detected by mNGS

**Figure 2:**
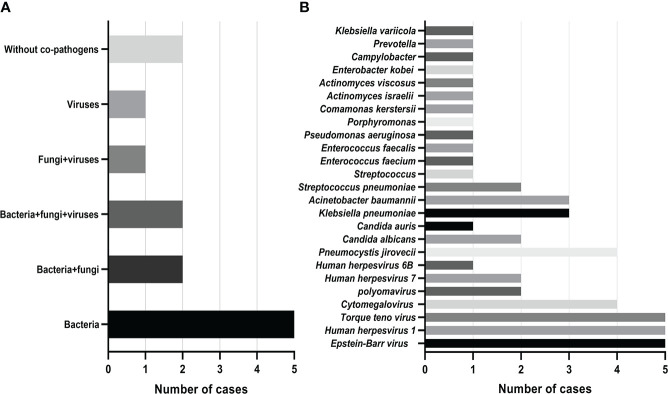
Mixed infections and co-pathogens in 13 non-neutropenic pulmonary aspergillosis cases identified using mNGS. **(A)** Number of pulmonary aspergillosis patients with mixed infections; **(B)** Number of pulmonary aspergillosis patients infected with various co-pathogens.

Mixed infections are common in non-neutropenic patients with pulmonary aspergillosis. Thirteen cases of putative mixed infections tested by mNGS are described in **Figure 2**. This method showed satisfactory performance in identifying co-pathogens (84.6%), among which *Aspergillus*-bacteria, *Aspergillus*-bacteria-fungi, and *Aspergillus*-bacteria-*P. jirovecii* (PJP)-virus were most common. *Aspergillus*-bacteria co-infections were identified by mNGS in 5 (38.5%) of 13 cases (**Figure 2A**). Closer inspection of the table shows that the most common bacteria were *Klebsiella pneumoniae* and *Acinetobacter baumannii*, the most common fungus was *P. jirovecii*, and the most common viruses were *Epstein–Barr virus*, *human herpesvirus 1*, and *Torque teno virus* (**Figure 2B**). This indicates that the prevention and treatment of mixed infections in pulmonary aspergillosis patients should be strengthened.

### Impact of mNGS on antimicrobial therapy of pulmonary aspergillosis in non-neutropenic patients

**Table 3** shows records of antimicrobial treatment for *Aspergillus* and other pathogens from 12 non-neutropenic patients with pulmonary aspergillosis during hospitalization. The most evident finding that emerged from the analysis is that based on the mNGS results, antimicrobial therapy was altered in 91.7% of patients, 58.4% had the antimicrobial agents or spectrum adjusted, 50% were started with anti-*Aspergillus* drugs, 16.7% were started with trimethoprim-sulfamethoxazole (TMP-SMZ), and 25% were started with anti-viral drugs.

**Table 3:** Impact of mNGS on antimicrobial therapy of pulmonary aspergillosis in non-neutropenic patients.

Modifications	Pulmonary aspergillosis patients (n = 12)
No change	1 (8.3%)
Remove 1 antimicrobial agent type	3 (25%)
Increase antimicrobial spectrum	2 (16.7%)
Reduce antimicrobial spectrum	2 (16.7%)
Add anti-*Aspergillus* drugs	6 (50%)
Add TMP-SMZ	2 (16.7%)
Add anti-viral drugs	3 (25%)

TMP-SMZ, trimethoprim-sulfamethoxazole.

### Detection of aspergillus in BALF and blood samples by mNGS

In this study, simultaneous mNGS of BALF and blood samples was performed in three patients who were suspected to have pulmonary aspergillosis. In two of the three patients, the stringent mapped read number (SMRN) of *Aspergillus* was detected in BALF but not in blood, and the clinical composite diagnosis was non-pulmonary aspergillosis. In summary, the diagnosis of *Aspergillus* detected in blood samples, which can be used as a supplement to BALF samples, showed higher specificity than that in BALF samples.

## Discussion

We performed a retrospective study to evaluate the diagnostic ability of mNGS in non-neutropenic patients with pulmonary aspergillosis. We showed that mNGS had satisfactory sensitivity in diagnosing and was useful in identifying co-pathogens in pulmonary mixed infections and directing modifications of antimicrobial treatment.

*Aspergillus* is a common opportunistic pathogen that can be fatal in certain conditions (such as organ transplantation, granulocytic, HIV-infected, and tumors). Currently, there is an increase in the occurrence of pulmonary aspergillosis in the non-neutropenic population owing to the increasing occurrence of non-specific factors such as COPD, diabetes, and immunosuppressive therapy.

Microbiological diagnosis of pulmonary aspergillosis is the ‘‘rate-limiting’’ step in achieving prompt treatment initiation and improving the prognosis of patients. Thus far, fungal smear and culture, serum (1,3)-β-D-glucan (G) or galactomannan (GM) tests, PCR, and *Aspergillus*-specific lateral flow device tests (LFD) (Jenks et al., 2019) are used in the microbiological analysis of pulmonary aspergillosis. Recent studies have shown that the polymorphism of PTX3 (which is a soluble pattern recognition receptor) is associated with increased susceptibility to invasive aspergillosis (Kang et al., 2020). However, these methods have certain limitations. Although traditional etiological culture methods help provide fungal drug sensitivity information, the clinical positivity rate is still low and fails to differentiate between contamination and colonization. Meanwhile, the sensitivity of the serum GM test in non-neutropenic patients is low and is affected by the use of antibiotics and intravenous immunoglobulins.

To date, mNGS has been clinically used. There are only few studies that have investigated the association between mNGS and the diagnosis of pulmonary aspergillosis (He et al., 2019; Zhang et al., 2020; Chen et al., 2021; Ma et al., 2022). In these studies, Chen et al. (Chen et al., 2021) and Zhang et al. (Zhang et al., 2020) reported the application of NGS in diagnosing co-infection of *Aspergillus* and *P. jirovecii*, which supports the results of this study. Additionally, Zhang et al. (Zhang et al., 2020) and He et al. (He et al., 2019) reported that patients with pulmonary aspergillosis have complications such as corticosteroid-treated dermatomyositis, COPD, and asthma, which is consistent with our results.

mNGS, as a new microbial diagnostic method, can accurately distinguish species and has high value for the diagnosis of *Aspergillus*. Therefore, our research concentrated on its diagnostic value, and we revealed that the sensitivity of mNGS is significantly higher than that of conventional etiological methods and serum (1,3)-β-D-glucan, but with relatively low specificity. Another notable benefit of mNGS over other microbiological tests is the wide spectrum of pathogen identification, which enables the detection of mixed infections with a single run of non-neutropenic pulmonary aspergillosis patient samples. Therefore, patients with mixed infections might benefit from this technique.

The unbiased broad-spectrum detection of mNGS could further guide effective antimicrobial treatment. In the present study, 50% of pulmonary aspergillosis patients did not receive anti-*Aspergillus* drugs until the report of mNGS results, demonstrating that mNGS is useful for the diagnosis and proper treatment of pulmonary aspergillosis. Triazoles are the only oral drugs with anti-*Aspergillus* activity, while itraconazole and voriconazole are considered first-line drugs (Alastruey-Izquierdo et al., 2018). These findings imply that an earlier application of mNGS can guide a rapid and accurate diagnosis of pulmonary aspergillosis. This new method is expected to become a new gold standard with high therapeutic efficiency after diagnosis.

Notably, the mNGS of *Aspergillus* in BALF samples sometimes gives a false-positive result. This could be due to several factors, which include: (a) contamination of the microbial genome from the environment or body flora and (b) high host genome background and low microbial biomass of the true pathogens (Chen et al., 2020). mNGS of *Aspergillus* in blood samples has a higher specificity. A possible explanation is that healthy human blood is sterile. Meanwhile, the consistency of mNGS detection of *Aspergillus* in BALF and blood samples suggests that *Aspergillus* may be transmitted from the lungs to the bloodstream in patients with pulmonary aspergillosis (Jiang et al., 2021). In contrast, one study showed that the diagnostic value of BALF GM detection in non-neutropenic patients is superior to that of serum GM detection (Zhou et al., 2017).

The present study had several limitations. Firstly, it was a single-center retrospective study. Thereby, intrinsic bias is inevitable. In addition, the diagnostic performance of mNGS was not compared with that of the GM test, which our hospital does not routinely perform. Therefore, further studies that consider these variables are needed. Moreover, owing to the lack of general agreement on interpreting mNGS results, it is difficult to determine whether microbes reported by mNGS are significant clinical pathogens or colonized microbes, which must be categorized based on a thorough examination of clinical characteristics, laboratory tests, and parameters. Consequently, our study further analyzed the correlation between SMRN of *Aspergillus* detected by mNGS and the results of other clinical tests in 12 diagnoses of pulmonary aspergillosis cases. No significant differences between the SMRN of *Aspergillus* in BALF or blood samples and clinical data were observed.

Finally, there are certain drawbacks associated with the use of mNGS. While it takes 24–48 h to complete all processes, a single run of mNGS costs more than traditional detection methods. The high costs and requirements of mNGS are a barrier to the widespread use (such as blood routine examination). However, mNGS is a powerful tool for the unexplained and severe pneumonia, such as pulmonary aspergillosis. In addition, the generalizability of mNGS is subject to certain limitations. For instance, the cell wall of *Aspergillus* is thick and nucleic acids are difficult to release, which causes a false-negative result. Furthermore, many *Aspergillus* genome databases are incomplete, and the public database is contaminated with human *Aspergillus* sequences. Consequently, mNGS can only be used as an auxiliary diagnostic index and not as a basis for etiological diagnosis. In addition to the study of its diagnostic value, greater efforts are needed for further dynamic testing of pulmonary aspergillosis in non-neutropenic patients using mNGS during the entire disease course. It is thus worthwhile to explore its value in predicting anti-*Aspergillus* treatment efficacy (Hagiwara et al., 2014).

All the studies reviewed thus far, however, suffer from the fact that the value of mNGS for the diagnosis of pulmonary aspergillosis in non-neutropenic patients remains poorly studied; further multicenter prospective studies with large sample sizes are needed.

## Conclusions

The following conclusions were drawn from this study: mNGS is a useful and unbiased diagnostic method for pulmonary aspergillosis in non-neutropenic patients and should be adopted as soon as possible. It has high sensitivity in identifying pulmonary aspergillosis and outperforms other ways in detecting mixed infections and adjusting antimicrobial treatment. This new method has high potential to be adopted as a gold standard.

## Data availability statement

The data analyzed in this study is subject to the following licenses/restrictions: All data supporting this study are reasonably available from the corresponding author. Requests to access these datasets should be directed to Hao Tang, tanghao_0921@126.com.

## Ethics statement

The studies involving human participants were reviewed and approved by Medical Ethics Committee, Shanghai Changzheng Hospital. The patients/participants provided their written informed consent to participate in this study. Written informed consent was obtained from the individual(s) for the publication of any potentially identifiable images or data included in this article.

## Author contributions

SB and HT conceived the project. SB, HS, YC, and CZ collected cases. HS and YC analyzed and interpreted patient data. SB and CZ wrote the manuscript. All authors have read and approved the final manuscript. SB, HS, YC, and CZ contributed equally to this work and share first authorship.

## Funding

The research was sponsored by “Shuguang Program” supported by Shanghai Education Development Foundation and Shanghai Municipal Education Commission (20SG38), Shanghai Municipal Science and Technology Committee of Shanghai Outstanding Academic Leaders Plan (20XD1423300), and General Program of National Nature Science Foundation of China (No. 82070036).

## Acknowledgments

The authors would like to thank all patients for participating in this study. The authors also thank the BGI (Shanghai, China) for their helpful technical support.

## Conflict of interest

The authors declare that the research was conducted in the absence of any commercial or financial relationships that could be construed as a potential conflict of interest.

## Publisher’s note

All claims expressed in this article are solely those of the authors and do not necessarily represent those of their affiliated organizations, or those of the publisher, the editors and the reviewers. Any product that may be evaluated in this article, or claim that may be made by its manufacturer, is not guaranteed or endorsed by the publisher.
